# Immunosuppressive cells in oncolytic virotherapy for glioma: challenges and solutions

**DOI:** 10.3389/fcimb.2023.1141034

**Published:** 2023-05-10

**Authors:** Junfeng Liu, Raziye Piranlioglu, Fei Ye, Kai Shu, Ting Lei, Hiroshi Nakashima

**Affiliations:** ^1^ Harvey Cushing Neuro-Oncology Laboratories, Department of Neurosurgery, Brigham and Women’s Hospital, Harvard Medical School, Boston, MA, United States; ^2^ Department of Neurosurgery, Tongji Hospital, Tongji Medical College, Huazhong University of Science and Technology, Wuhan, China

**Keywords:** oncolytic virus, glioma, immunosuppressive, glioblastoma, macrophages, microglia, MDSCs, tumor microenvironment

## Abstract

Glioblastoma is a highly aggressive form of brain cancer characterized by the abundance of myeloid lineage cells in the tumor microenvironment. Tumor-associated macrophages and microglia (TAM) and myeloid-derived suppressor cells (MDSCs), play a pivotal role in promoting immune suppression and tumor progression. Oncolytic viruses (OVs) are self-amplifying cytotoxic agents that can stimulate local anti-tumor immune responses and have the potential to suppress immunosuppressive myeloid cells and recruit tumor-infiltrating T lymphocytes (TILs) to the tumor site, leading to an adaptive immune response against tumors. However, the impact of OV therapy on the tumor-resident myeloid population and the subsequent immune responses are not yet fully understood. This review provides an overview of how TAM and MDSC respond to different types of OVs, and combination therapeutics that target the myeloid population to promote anti-tumor immune responses in the glioma microenvironment.

## 1 Introduction

### 1.1 Oncolytic virotherapy for glioma: current challenges

Glioma, the most common primary central nervous system (CNS) tumor in adults, is characterized by aggressive clinical-biological behavior, with low-grade gliomas eventually progressing to CNS WHO grade 3/4 gliomas and resulting death (Claus et al., 2015). Glioblastoma (Louis et al., 2021), the most lethal type of glioma, is resistant to conventional therapies and shows invasive, intratumoral heterogeneity and stem-like phenotypic plasticity (Liu et al., 2018; Perus and Walsh, 2019; Prager et al., 2020; Hutoczki et al., 2021; Nicholson and Fine, 2021; Knudsen et al., 2022). The median survival time is less than two years despite multimodality treatment options, such as maximum safe surgical resection, chemotherapy, radiotherapy, and other new treatment strategies (Tan et al., 2020). The blood-brain barrier (BBB) limits therapeutic drug options for glioma patients by actively blocking the influx of potentially effective pharmaceutical small molecules and antibodies from peripheral routes (Sarkaria et al., 2018; Luo and Shusta, 2020). BBB also maintains immune homeostasis in the brain by filtering leukocytes from attempts of peripheral adaptive immune cells into the brain to protect vulnerable neuronal cells from inflammation and autoimmunity (Muldoon et al., 2013). However, glioma takes advantage of this immunological privilege to escape from immunosurveillance and creates an immunosuppressive tumor microenvironment. This results in further evolving immune evasion mechanisms and neutralizing current immune checkpoint inhibitor (ICI) therapies as a “cold” tumor (Lim et al., 2018; Labani-Motlagh et al., 2020).

Oncolytic virotherapy is an emerging treatment modality that holds great promise for the treatment of cancer. Genetically engineered viruses can selectively destroy cancer cells while sparing healthy cells through altering viral infection, replication or both (Chiocca, 2002; Twumasi-Boateng et al., 2018). In addition to their direct lytic toxicity, OVs have been shown to induce systemic anti-tumor immune responses. Some oncolytic viruses are also capable of naturally targeting tumor stroma components, including cancer-associated fibroblasts and tumor vasculature, complicating their anti-tumor mechanisms (Toro Bejarano and Merchan, 2015; Everts et al., 2020). Moreover, OVs can trigger immunogenic cell death (ICD), which involves the release of tumor-associated antigens (TAA), pathogen- or damage-associated molecular patterns (PAMPs or DAMPs), and inflammatory cytokines and chemokines (Ma et al., 2020; Hofman et al., 2021). Additionally, genetic engineering can be used to enhance OVs’ anti-tumor immunity by expressing immune stimulant factors, leading to potent and long-lasting adaptive immunity, potentially transforming the tumor microenvironment from “cold” to “hot” (Friedman et al., 2021).

Currently, several OVs, including Herpes Simplex Virus (HSV), Adenovirus (AdV), Reovirus, Newcastle Disease Virus (NDV), Poliovirus, and others, are under evaluation in preclinical studies and clinical trials for gliomas with promising results (**Table 1**) (Rius-Rocabert et al., 2020; Lu et al., 2021; Shoaf and Desjardins, 2022). A recent development in Japan has received conditional and time-limited approval for G47Δ, an HSV1-based OV, for patients with glioblastoma (Shoaf and Desjardins, 2022). Nevertheless, glioblastoma remains an incurable cancer type, and OV monotherapy faces significant challenges (Zhang and Liu, 2020). First, the administration of a single dose of intratumoral OV may not be sufficient to exert anti-tumor effects due to uneven spread, lack of persistence, and rapid clearance of virus particles (Moaven et al., 2021; Shoaf and Desjardins, 2022). Second, the immunosuppressive microenvironment in gliomas hinders T cell activation and induces exhaustion. Third, innate immunity activated by OV therapy subsequently inhibits the replication and spread of OV. Fourth, some OVs have the potential to promote angiogenesis, which can support tumor growth and migration (Kurozumi et al., 2008). Finally, despite demonstrating tolerable safety in most glioma clinical trials (Shalhout et al., 2023), the adverse events and long-term complications of OVs still require continuous attention for large-scale application. Therefore, this review article aims to provide the current status of OV therapy, including its impact on the immunosuppressive glioma microenvironment and therapeutic limitations, to guide future directions for research and development in the field.

**Table 1:** Clinical trials of oncolytic virotherapy for gliomas.

Virus name	Genetic modifications	Administration approach	Combination therapies	Tumors	Phase	Country		Year		References
Adenovirus (Ad)										
Ad-TD-nsIL12	nsIL12, deletions in E1ACR2, E1B19K and E3gp19K),	Single i.t.		DIPG	1	China		2023		NCT05717712; NCT05717699
DNX-2401 (Delta-24-RGD; tasadenoturev)	E1A deletion, an RGD-fiber	an infusion in the cerebellar peduncle	RT	NaiveDIPG	1	Spain		2017		NCT03178032 (Lang et al., 2018; Perez-Larraya et al., 2022)
	Loaded with MSC	ESIA infusions	Therapeutic Conventional Surgery	Recurrent HGG	1	USA		2019		NCT03896568 (Chen et al., 2022)
		CED to infiltrated brain		Recurrent GBM	1/2	Netherlands		2010		NCT01582516
		i.t.	Pembrolizumab, CAPTIVE/KEYNOTE-192	Recurrent GBM or GBM	2	USA		2016		NCT02798406
		Single i.t.	IFN-γ	Recurrent GBM or GBM	1	USA		2014		NCT02197169
		i.t. or resected cavity	TMZ	Recurrent GBM	1	Spain		2013		NCT01956734
CRAd-S-pk7	survivin promoter-E1A, fiber-pk7 loaded on NSC	Resected cavity	TMZ + RT	Newly Diagnosed HGG	1	USA		2017		NCT03072134 (Fares et al., 2021)
		i.c.	Surgical resection	Recurrent HGG	1	USA		2023		NCT05139056
DNX-2440		i.t.		first or second recurrence GBM	1	Spain		2018		NCT03714334
ICOVIR-5	Loaded on Allogenic MSC	weekly infusion	DIPG: RT, Medulloblastoma: monotherapy	Newly Diagnosed DIPG or Medulloblastoma	1/2	Spain		2021		NCT04758533
Ad5-yCD/mutTKSR39rep-ADP	yeast cytosine deaminase (yCD)/mutant (SR39) with HSV-1 TK (yCD/mutTK(SR39)), adenovirus death protein (ADP) gene	a single intratumoral injection	fractionated stereotactic radiosurgery (fSRS), oral 5-fluorocytosine (5-FC) and valganciclovir (vGCV)	Recurrent high-grade astrocytoma	1	USA		2022		NCT05686798
HSV-1/2										
G207	deletions of both γ_1_34.5 and a lacZ:UL39	i.t.		Recurrent Malignant Glioma		USA		2001		NCT00028158 (Markert et al., 2000)
		MRI-guided single infusion	RT (5Gy)	Recurrent HGG in Children	2	USA		2023		NCT04482933
				Recurrent or Refractory Cerebellar Brain Tumors in Children	1	USA		2019		NCT03911388
G47delta	α47 deletion and α47 promoter-US11 gene in G207	6 or more i.t. injection		Residual or recurrent GBM	1/2	Japan		2009, 2014		UMIN000002661 (UMIN-CTR), UMIN000015995 (UMIN-CTR), (Todo et al., 2022)
rQNestin34.5v.2 (CAN-3110)	γ_1_34.5 and UL39 gene deletion, nestin promoter- γ_1_34.5 gene insertion	MRI-guided i.t.	Cyclophosphamide (2^nd^ arm only)	Recurrent or progressive brain tumor	1	USA		2017		NCT03152318 (Chiocca et al., 2020)
C134		Multiple i.t.		Recurrent Malignant Glioma	1	USA		2019		NCT03657576
M032	γ_1_34.5 deletion, hIL-12 gene insertion	Single infusion		Recurrent Malignant Glioma	1	USA		2013		NCT02062827
			Pembrolizumab	Recurrent/Progressive and Newly Diagnosed Malignant Glioma	1/2	USA		2022		NCT05084430
MVR-C5252	IL-12 and Anti-PD-1 insertion	a single i.t.		Recurrent or Progressive GBM	1	USA		2023		NCT05095441
OH2	ICP34.5 and ICP47 gene deletion in HSV-2, hGM-CSF gene insertion	administered in tumor cavity by Ommaya reservoir injection		Recurrent CNS tumor (Phase 1), Recurrent GBM (Phase 2)	1/2	China		2021		NCT05235074
Other types of oncolytic viruses										
H-1PV (ParvOryx)	H-1 protoparvovirus	Three doses via i.t. or i.v., followed by i.c. into the walls of the resection cavity		Progressive Primary or Recurrent GBM	1/2	Germany		2011		NCT01301430 (Geletneky et al., 2012; Geletneky et al., 2017)
REOLYSIN®		single i.t. infusion over 72 hours		Recurrent Malignant Gliomas	1/2	USA		2006		NCT00528684
PVSRIPO	a live attenuated poliovirus type 1 (Sabin) vaccine with its cognate IRES with that of human rhinovirus type 2	i.t. with CED		Recurrent WHO grade 4 malignant glioma	2	USA		2017		NCT02986178
		i.t. with CED		Recurrent Malignant Glioma (WHO grade 3 or 4) in Children (12-21 yr)	1b	USA		2017		NCT03043391
		i.t. with CED		Recurrent WHO Grade 4 malignant glioma	1	USA		2012		NCT01491893 (Desjardins et al., 2018)
TG6002	J2R, the I4L gene deletions in vaccinia, FCU1 insertion	weekly i.v. infusions at days 1, 8 and 15	5-flucytosine (5-FC)	Recurrent Glioblastoma	1/2	France		2017		NCT03294486

i.t., intratumoral injection; i.c., intracerebral injection; i.v., intravenous injection; DIPG, Diffuse Intrinsic Pontine Gliomas; ESIA, endovascular super-selective intra-arterial; CED, convection-enhanced delivery; TMZ, temozolomide; RT, radiotherapy; Pem, Pembrolizumab; HGG, high-grade glioma; GBM, glioblastoma; IRES, internal ribosome entry site; CNS, central nervous system; WHO, world health organization; MSC, mesenchymal stem cell; NSC, neural stem cell; RGD, arginine-glycine-aspartame.

### 1.2 The presence of immunosuppressive cells in the tumor microenvironment of gliomas

The interplay between immunosuppressive cells and oncolytic viruses (OVs) in glioma therapy is complex and involves dynamic and multifaceted virus-induced immune responses. The involvement of immunosuppressive cells in OV therapy is not straightforward since they can either act as foes or friends. Various immune cell subsets, such as tumor-associated microglia/macrophages (TAMs), myeloid-derived suppressor cells (MDSCs), regulatory T cells (Tregs), and tumor-associated neutrophils (TANs), mediate immunological suppression that contributes to the complex outcomes observed in OV therapy. Tumor cells attract these cells to the microenvironment and alter their functions and phenotypes by secreting chemoattractants, such as MIC-1 (Wu et al., 2010), MCP-1 (Roesch et al., 2018), GM-CSF (Horikawa et al., 2020), S100A8/9 (Gabrilovich and Nagaraj, 2009) and CCL2 (Chang et al., 2016), to create an immunosuppressive milieu that aids in evading anti-tumor immunity.

The immunosuppressive cells present in the glioma microenvironment consist of brain tissue-resident and peripherally derived immune cells. Microglia are the primary resident immunosuppressive cells in the brain, accounting for 13-34% of the tumor mass (Gieryng et al., 2017). Macrophages are main peripheral immunosuppressive cells, accounting for 5-12% of the tumor mass (Gieryng et al., 2017). Microglia and macrophages comprise up to 30-50% of the cells in the glioma microenvironment, while MDSCs are the second largest subpopulation of immunosuppressive cells in the glioma microenvironment after TAMs, accounting for 5-8% of glioma mass (Hambardzumyan et al., 2016; Gieryng et al., 2017), but some literatures suggest a larger population (Gabrusiewicz et al., 2016; Kamran et al., 2017). Tregs are rare and account for only 0.3% of tumor mass (Thomas et al., 2015). TANs are mature neutrophils in the glioma microenvironment, accounting for a smaller proportion, and are often confused with polymorphonuclear (PMN)-MDSCs (Bronte et al., 2016). Despite their small proportion, both Tregs and TAN play important roles in immune modulation. TAMs and MDSCs are particularly significant in orchestrating an immunosuppressive microenvironment in glioma, and this review will focus mainly on their immunosuppressive functions in glioma virotherapy.

## 2 Tumor-associated microglia/macrophages

### 2.1 The role of TAMs in the immunosuppressive microenvironment of gliomas

Tumor-associated macrophages (TAMs) are a heterogeneous population of immune cells that play a dominant immunosuppressive role in the glioma microenvironment, comprising up to 50% of the tumor mass (Hambardzumyan et al., 2016; Twumasi-Boateng et al., 2018). TAMs include brain-resident microglia and monocyte-derived macrophages with the relative composition of these cells depending on various factors such as genotype, grade, progression stage, and spatial distribution of the tumors in the brain (Friebel et al., 2020; Andersen et al., 2022). In the normal brain, microglia are the primary myeloid cells as the resident macrophages in the central nervous system, while peripheral macrophages are rarely seen (DePaula-Silva et al., 2019). In gliomas carrying mutations in genes encoding isocitrate dehydrogenase 1 (IDH1) or 2 (IDH2), microglia-derived TAMs are more dominant compared to wild-type gliomas (Friebel et al., 2020). Moreover, the density of bone marrow-derived macrophages increases with tumor progression and the glioma grades (Sorensen et al., 2018). The spatial distribution of microglia and peripheral macrophages is distinct within the glioma microenvironment. Microglia preferentially reside in tumor-adjacent parenchymal regions, while bone marrow-derived macrophages are more abundant inside the tumor, particularly in peri-necrotic and peri-vascular areas (Landry et al., 2020; Yin et al., 2022). Microglia specific molecular markers such as TMEM119 and P2RY12 are applicable to distinguish microglia from peripheral macrophages (van Wageningen et al., 2019; Mercurio et al., 2022). Despite their differences in ontogeny and spatial distribution, microglia and peripheral macrophages share similar functions in the glioma microenvironment and are referred as single cell cluster, i.e., TAMs (Blitz et al., 2022; Rao et al., 2022).

TAMs have two distinct phenotypes: resting (or non-activated) and polarized (or activated) (Li et al., 2021), with the latter being further classified into two functionally distinctive types known as M1 and M2 phenotypes (Mills et al., 2000; Yunna et al., 2020). The M1 type is the classically activated antitumor phenotype, while the M2 type is the alternatively activated pro-tumor phenotype. The concept of M1-M2 classification was initially proposed by Hill et al. based on observations of activated macrophages resembling Th1-Th2 polarization of T cells (Mills et al., 2000). M1-like TAMs are induced by interferon-γ (IFN-γ), tumor necrosis factor α (TNF-α), and granulocyte-macrophage colony-stimulating factor (GM-CSF) and are involved in pro-inflammatory responses and antigen presentation with costimulatory molecules such as CD80 and CD86 (de Sousa et al., 2016). M2-like TAMs are stimulated by interleukin (IL)-10 and transforming growth factor β (TGF-β) and involved in anti-inflammatory responses with overexpressed surface proteins such as CD206, CD204 and CD163 (Laviron and Boissonnas, 2019; Yunna et al., 2020). Although the activation state of TAMs usually changes dynamically and continuously between M1 and M2 (Martinez and Gordon, 2014), there is no clear boundary between the two polarized states, especially *in vivo* conditions. The M1-M2 model became obsolete after extensive studies with widely applicable single-cell and spatial technologies such as single-cell RNA and ATAC sequencing with spatially resolved profiling (Deng et al., 2022a; Deng et al., 2022b). Recently, TAM subpopulation is redefined as described elsewhere (Ma et al., 2022; Pittet et al., 2022). However, M1/M2 dichotomy facilitates research and communications in the field of macrophages. The terminology of M1-M2 is frequently used in papers on OV therapy; thus, we used the M1 and M2 terms to respect descriptions of original papers through this review.

The predominant phenotype of tumor-associated macrophages (TAMs) in the tumor microenvironment during glioma onset and early stages remains unclear (Kennedy et al., 2013). Nevertheless, studies indicate that M2 TAM infiltration increases with glioma progression (Mahlbacher et al., 2018; Yin et al., 2020; Ghosh et al., 2022). Glioma cell proliferation, including glioma stem cells (Yi et al., 2011), results in the secretion of cytokines and chemokines such as MIC-1, periostin, IL-33, and MCP-1 (Wu et al., 2010; Zhou et al., 2015; Roesch et al., 2018; De Boeck et al., 2020), which recruit blood-borne monocytes and macrophages to the tumor site, polarizing them into M2 TAMs. M2 TAMs play a significant role in various malignant biological behaviors, such as tumor proliferation, invasion, angiogenesis, and stemness maintenance (Hambardzumyan et al., 2016; Roesch et al., 2018; Zhu et al., 2018; Geraldo et al., 2021; Yan et al., 2021; Yeini et al., 2021). They also contribute to the immunosuppressive microenvironment of gliomas by releasing soluble factors, metabolites, or direct cell-cell interactions (Roesch et al., 2018). M2-like TAMs directly impair effector T cells activation or induce apoptosis by binding to T cells inhibitory receptors, such as CTLA-4 and PD-1 (Seliger et al., 2008; Saha et al., 2017) or death receptors, such as FAS and DR5 (Zhu et al., 2019). M2-like TAMs also release cytokines, such as TGF-β and IL-10 and promote the production of metabolites, such as indoleamine 2,3-dioxygenase (IDO) and kynurenine, inhibiting functions of T cells, NK cells and DCs (Roesch et al., 2018; Herrera-Rios et al., 2020). Furthermore, M2 TAMs promote the recruitment of other immunosuppressive cells like MDSCs and Tregs *via* cytokines and chemokines, such as IL-10, TGF-β, IL-4, IL-6, CCL2, CCL5, and CCL20 (Zhou et al., 2020). Lastly, M2 TAMs promote immune evasion of glioma cells by binding to the “don’t eat me” signal molecule CD47, which is overexpressed on the cell surface of gliomas (Zhang et al., 2016). In summary, TAMs play a dominant immunosuppressive role in the glioma microenvironment, with M2-like TAMs involved in various malignant behaviors. Understanding the function of TAMs in glioma progression can aid in developing effective OV therapies for gliomas.

### 2.2 The effects of OVs on TAMs

**Figure 1:**
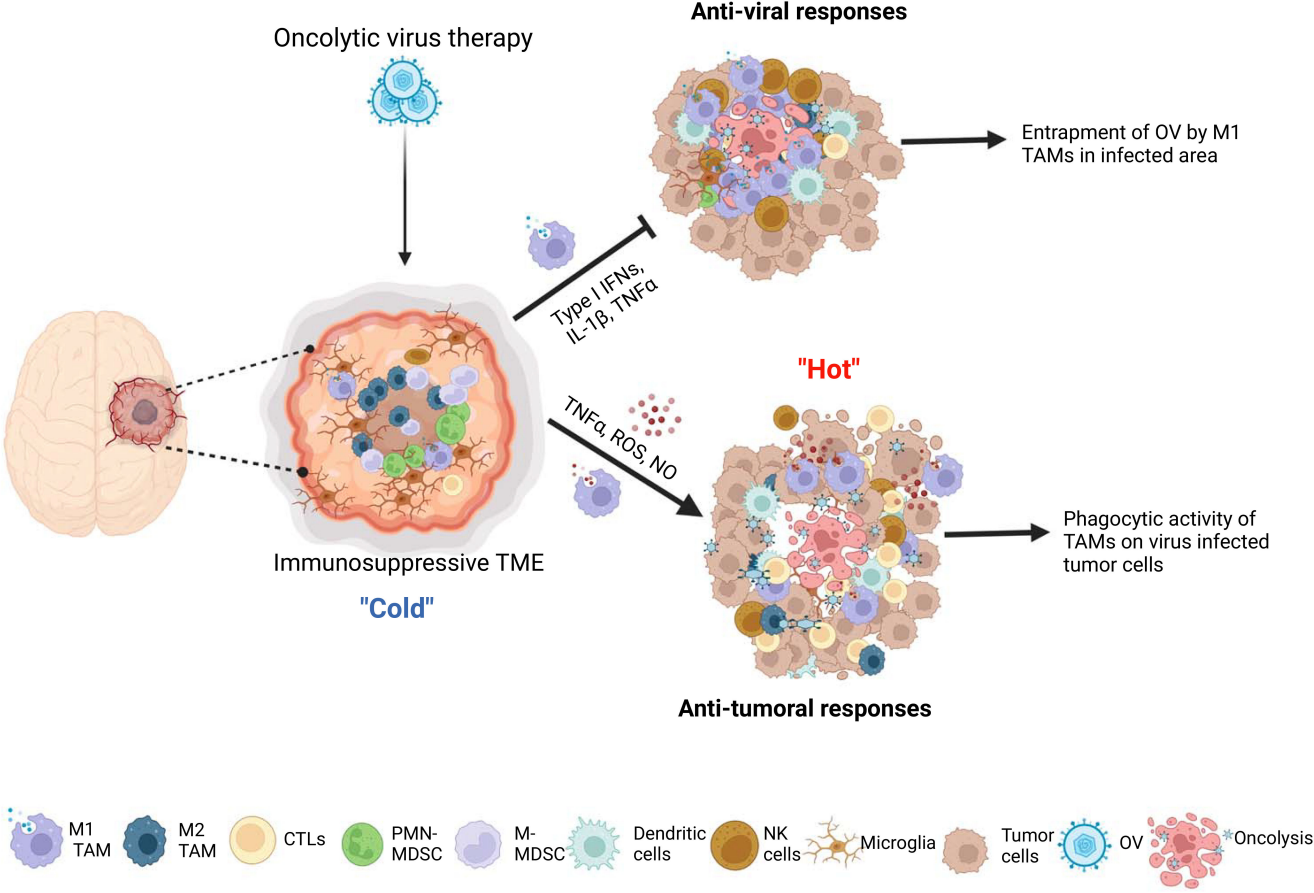
The interactions between microglia/macrophages and oncolytic viruses (OVs) in gliomas. The infection glioma cells with OVs lead to recruitment more microglia and peripheral macrophages to the OV administration site and polarization of preexisting M2 type microglia/macrophages to M1 type tumor associated macrophages (TAMs). These M1 TAMs form a physical barrier surrounding the OVs-infected area to hinder the replication and spread of viruses. On one hand, M1 TAMs exert anti-viral functions through secretion of anti-viral cytokines, by recruiting other anti-viral immune cells and by direct phagocytosis of viral particles. On the other hand, these cells exert anti-tumor activity through production of tumor-killing factors, by inducing the recruitment of other anti-tumor immune cells, such as NK cells and dendritic cells, and, more importantly, acting as antigen presenting cells to elicit adaptive anti-tumor immune responses.

Crosstalk between OV-infected tumor and TAM results in complex outcomes in OV therapy. Following administration, OVs modulate the tumor microenvironment by attracting bone marrow-derived macrophages and brain resident microglia to the OV-injected tumor site through the release of chemoattractants by OV-infected tumor cells, such as CCL2 and CCN1 (Parker et al., 2005; Thorne et al., 2014; Meisen et al., 2015). M1-like macrophages are recruited in the early stage of virus infection, and play a crucial role in virus clearance, while M2-like macrophages contribute to wound healing and tissue repair in the late stage of infection (Clements et al., 2017). However, current research does not provide evidence of a shift from M1 to M2 during OV infection at tumors. OV action may recruit and polarize non-activated monocytes into M1-like macrophages (Meisen et al., 2015; Clements et al., 2017) and promote the switching from M2 to M1 phenotypes of pre-existing tumor-associated macrophages (TAMs) in the tumor microenvironment (van den Bossche et al., 2018; Lee et al., 2019; Hofman et al., 2021; Blitz et al., 2022) (**Figure 1**).

### 2.3 The negative impacts of TAMs on OVs

Effectiveness of oncolysis ability to destroy cancer cells can be attenuated through immune responses elicited by M1-like TAM, which OV action trigger to recruit. They respond to the viral infection and play a role in the clearance of virus-infected cells by stimulating inflammation and anti-viral reaction. During the lytic cycle of virus infection in tumors, macrophages are rapidly recruited to virally infected tumors and activated to aid in virus clearance, primarily through phagocytosis (Clements et al., 2017; Nikitina et al., 2018). When OV is intratumorally administered to gliomas, TAM accumulate around the OV injection site within the tumor mass and form physical barriers to limit the spatial distribution of virions over the injected areas (Blitz et al., 2022). Microglia and macrophages are capable of uptaking oncolytic herpes simplex virus (oHSV) particles in glioma. However, oHSV replication in these cells is typically inhibited due to the activation of the intrinsic and intracellular innate immunity through STAT1-interferon axis signaling pathways (Delwar et al., 2018). Consequently, tumor-associated macrophages (TAMs) phagocytose OV, which limits the tumor-killing effects of oncolytic viruses (Fulci et al., 2007; Kober et al., 2015) (**Figure 1**). Moreover, OV injection preferentially recruits M1-like TAMs rather than M2-like TAMs, which prevent the infection and dissemination of OVs (Blitz et al., 2022). TAMs are recruited to the OV injection site and form physical barriers that restrict the spread of virions within the tumor. Unlike oHSV, VV-based OVs are capable of infecting TAMs, but their replication is inhibited by early gene-induced apoptosis in these cells, which is not typically observed in permissive tumor cells (Humlova et al., 2002; Kober et al., 2015). A mathematical modeling and computational approach proposed that the susceptibility of macrophages to OVs is influenced by their polarization (Almuallem et al., 2020). Specifically, M2-like macrophages are more susceptible to oncolytic vesicular stomatitis virus (oVSV) infection than M1 macrophages, according to a recent study (Polzin et al., 2020). Additionally, the migration of oVSV-infected M2 macrophages towards the hypoxic region of the tumor has been shown to facilitate OV dissemination (Almuallem et al., 2020). However, in response to OV injection, M1-like TAMs are selectively recruited instead of M2-like TAMs, which impedes OV infection and dissemination (Blitz et al., 2022).

Macrophages serve as the primary immune response against viral infections and secrete anti-viral cytokines and chemokines, such as TNF-α, interferons, IL-1β, and IL-12. However, these immune responses can also attenuate the efficacy of OV therapy in the tumor microenvironment (Clements et al., 2017; Nikitina et al., 2018). The secretion of TNF-α by TAMs in response to OV therapy has been shown to be a crucial factor in inhibiting viral replication by inducing apoptosis in OV-infected glioma cells (Meisen et al., 2015; Yoo et al., 2019). This promotion of apoptotic cell death can lead to a decrease in viral infection or replication (Liskova et al., 2011; Kober et al., 2015). The M1-like phenotype of TAMs, which are predominantly found surrounding the injection site of OV, have the ability to produce interferons and eliminate viruses through a Type I interferon-dependent mechanism (Lang et al., 2010; Almuallem et al., 2020; Nikonova et al., 2020). Moreover, pro-inflammatory macrophages (M1 type) polarized by OV could recruit and activate other innate immune cells, such as natural killer cells (NK cells) and DCs, through secretion of chemokines and cytokines, thus further enhancing anti-viral innate immune response (Denton et al., 2016) (**Figure 1**).

### 2.4 Anti-tumor effects of TAMs on OV

Despite their negative impact on OV infection, OV-stimulated TAMs may have a positive impact on anti-tumor effects. A large number of studies have shown that M1-polarized microglia/macrophages can inhibit proliferation (Yin et al., 2017), invasion (Wang et al., 2021) and angiogenesis (Cui et al., 2018), as well as promote anti-tumor immune response (Hsu et al., 2020) in gliomas. After oncolytic virus treatment, the M2 pro-tumor TAMs that were previously present in the glioma microenvironment repolarize towards the M1 phenotype, which is known to have anti-tumor and pro-inflammatory properties (Meisen et al., 2015; Ma et al., 2022). Various types of tumors, including gliomas, have been shown to benefit from anti-tumor effects of TAMs in oncolytic virotherapy, regardless of the type of OV used (Meisen et al., 2015; van den Bossche et al., 2018; Hofman et al., 2021; Kim et al., 2021; Milenova et al., 2021). For example, M1 TAMs have the ability to generate soluble factors, including reactive oxygen species (ROS), nitric oxide (NO), TNF-α, and IL-1β, which can cause apoptosis, DNA damage, or cytotoxicity, leading to the direct killing of tumor cells (Pan et al., 2020; Aminin and Wang, 2021). M1 TAMs also have an indirect anti-tumor effect by recruiting and activating other immune cells, such as NK and T cells. NK cells are part of the innate immune system and have strong cytotoxic functions against tumor cells and other abnormal cells. M1 macrophages collaborate with NK cells to eliminate tumor cells (Aminin and Wang, 2021). M1-polarized macrophages, being one of the antigen-presenting cells (APCs), are capable of presenting tumor or virus-associated antigens to effector T cells, thereby triggering a vigorous adaptive anti-tumor immune response (Burke et al., 2020; Hofman et al., 2021). Multiple studies have demonstrated the anti-tumoral roles of TAMs in OV therapy for gliomas. A recent study shows an oncolytic IL-12-expressing HSV-1, G47Δ-mIL12, skewed TAMs to M1-like phenotype, and M1-like TAMs were further increased by triple therapy consisting of anti-CTLA-4, anti-PD-1, and G47Δ-mIL12 in the GSC-derived GBM models. The authors found that triple treatment significantly increased the cure rate of GBM-bearing mice. Still, TAMs depletion blocked the efficacy of triple therapy, indicating that TAMs, in part, play an indispensable role in the treatment of GBM with this triple OV immunotherapy (Saha et al., 2017). Similarly, Xu and colleagues reported that macrophages mediate the anti-tumor cytotoxicity of αCD47-IgG1-producing oncolytic HSV-1 in a preclinical model of GBM (Xu et al., 2021). Together, these findings demonstrate that TAMs within the glioma microenvironment upon OV therapy exhibit tumor-killing function and possess classical activated M1-type characteristics (**Figure 1**).

### 2.5 TAMs-targeted therapy in combination with OV therapy

**Figure 2:**
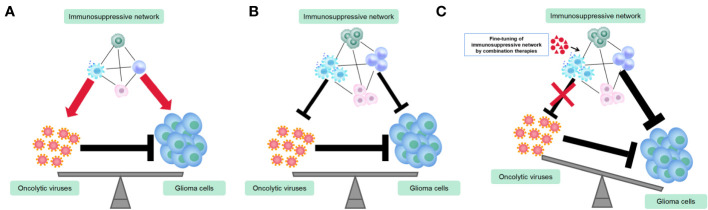
Utilization of combination therapies to fine-tune immunosuppressive networks in the glioma virotherapy. **(A)** Prior to or during the initial stage of oncolytic virus (OV) therapy, the immunosuppressive network exerts both pro-tumoral and pro-viral functions. **(B)** OVs recruit more immuosuppressive cells and alter their functions and phenotypes. Immunosuppressive network exerts both anti-tumoral and anti-viral functions. **(C)** Fine-tuning immunosuppressive network by combination therapies renders maximum anti-tumor immune responses and minimum anti-viral immune responses.

TAMs play a dual role in OV therapy for gliomas by inhibiting OV replication and spread while also enhancing OV’s tumor-killing efficacy (Blitz et al., 2022). Therefore, combining TAMs-targeting immunotherapy with OV therapy can be challenging to predict the outcomes. TAMs act as a link between innate and adaptive immunity in OV treatment and are involved in complex immune regulatory networks. To maximize the anti-tumor effects of TAMs and minimize their negative impact on OVs, OV therapeutic strategy must carefully consider the function of TAMs (**Figure 2**). Current strategies primarily aim to mitigate the adverse effects of TAM on replication and spread of OV. An earlier study has shown that the depletion of microglia/macrophages with clodronate liposomes (CL) significantly increases the oncolytic HSV titers in syngeneic GBM models (Fulci et al., 2007). Similarly, cyclophosphamide (CPA) enhances HSV replication and oncolysis in GBM-bearing animals by inhibiting the OV-induced infiltration of TAMs and the production of IFN-γ by NK cells (Fulci et al., 2006). A depletion of TAM population is also promising approach. Shi et al. showed that a CSF1R inhibitor (PLX3397) combined with oncolytic adenoviruses and anti-PD-1 significantly promoted tumor regression and extended survival, as compared to single or dual therapies, in colon cancer models by depleting TAMs (Shi et al., 2019). On the other hand, a different study has suggested that the depletion of peripheral macrophages using clodronate liposomes or TAMs using CSF1R inhibitor BLZ945 eliminates the effectiveness of triple therapy comprising oncolytic HSV, anti-CTLA-4, and anti-PD-1 in GBM models. This indicates that M1-polarized TAMs play a crucial role in tumor suppression within the context of OV therapy (Saha et al., 2017). Furthermore, the CSF1R inhibitor, PLX3397, showed no efficacy in a phase II clinical trial in recurrent GBMs (Butowski et al., 2016). The findings of these two studies, which employed TAMs depletion therapy in conjunction with OV therapy and immune checkpoint blockades (ICBs), yielded opposite conclusions, which could be attributed to the disparities in the virus type, CSF-1R inhibitors employed, and the tumor type. Moreover, as discussed earlier, the efficacy of TAMs depletion is contingent on the tumor type and the timing of treatment (O'Brien et al., 2021). Given that TAMs exhibit remarkable plasticity with dynamically shifting phenotype and function in response to the signals within the tumor microenvironment, the timing of administration for TAMs-depletion compounds or antibodies is crucial for OV therapy. As an example, administering CPA 48 hours before OV injection can improve OV therapy in gliomas, as it transiently suppresses innate immune responses mediated by TAMs and NK cells, thereby allowing OV to spread and lyse tumors (Fulci et al., 2006). Similarly, administering a CSF-1R inhibitor during the early stages of glioma has demonstrated greater efficacy in inhibiting tumor growth and inducing adaptive immune responses (O'Brien et al., 2021). As a result, when employing the TAMs-depletion strategy combined with OVs for glioma treatment, it is crucial to exercise caution in identifying an optimal and rational treatment paradigm.

Another strategy to enhance the replication and spread of OVs is inhibiting M1 TAMs functions. TGF-β is an inflammatory cytokine that suppresses innate and adaptive immune responses. Han and colleagues showed that the administration of a single dose of TGF-β before OV therapy could transiently suppress innate immune cells, including microglia, macrophages, and NK cells which restrict efficacy of OVs, boosting therapeutic responses in glioma (Han et al., 2015; Groeneveldt et al., 2020). TNF-α is a TAMs-secreted anti-viral cytokine that is essential in inhibiting the replication and spread of OVs. Multiple studies have shown that curbing TNF-α secretion in gliomas can effectively increase viral replication and spreads, thereby improving the anti-tumor effects in OV therapy (Meisen et al., 2015; Yoo et al., 2019).

While inhibition of M1 TAM function is one strategy to enhance the replication and spread of OVs, another approach is to cooperate with TAMs instead of fighting them. One promising approach for TAMs-targeted combination therapy is the use of oncolytic HSV-1 expressing a full-length anti-human CD47 IgG1, which blocks the CD47 “don’t eat me” signal expressed on the surface of tumor cells, enhancing the phagocytosis of tumor cells by macrophages and improving the tumor-killing effect of OV therapy (Xu et al., 2021).

In conclusion, TAMs play a crucial role in OV therapy for gliomas. Combining TAMs-targeting immunotherapy with OV therapy for gliomas can be challenging due to the dual role of TAMs, but optimizing timing and type of therapy can enhance OV replication and spread while minimizing negative impact of TAMs, with strategies including depletion or inhibition of M1 TAM function and cooperation with TAMs.

## 3 Myeloid-derived suppressor cells

### 3.1 The role of MDSCs in the immunosuppressive microenvironment of glioma

MDSCs are a diverse group of myeloid cells that include immature macrophages, dendritic cells, and granulocytes at different stages of differentiation, which are present in very low numbers in healthy tissues, making up only 0.5-2% of peripheral blood mononuclear cells (PBMCs) (Salemizadeh Parizi et al., 2021). Under pathological conditions such as cancer, inflammation, trauma, and pathogen invasion, the proportions of MDSCs notably increase. The frequencies of MDSCs in gliomas vary, but they are generally considered the second largest immunosuppressive population within the glioma microenvironment after microglia/macrophages (Kamran et al., 2017; Salemizadeh Parizi et al., 2021). MDSCs are classified into two subtypes in mice, namely polymorphonuclear (PMN)-MDSCs and monocytic (M)-MDSCs, whereas in humans, there is a third phenotype referred to as early-stage MDSCs. These subtypes are distinguished based on their phenotypic and morphological characteristics. Most studies have identified MDSCs by analyzing their expression of specific cell surface markers. In mice, pan-MDSCs are usually characterized as CD11b+Gr-1+ cells, while PMN-MDSCs and M-MDSCs are defined as CD11b+Ly6G+Ly6Clo cells and CD11b+Ly6G-Ly6Chi cells, respectively (Bronte et al., 2016). Human MDSCs are classified based on their molecular markers. M-MDSC has the phenotype CD11b+HLA-DR−CD14+CD15-, while PMN-MDSC is marked as CD11b+HLA-DR−CD14-CD15+ (Bronte et al., 2016). However, these surface markers are commonly used but insufficient to define MDSC subpopulation. Bronte et al. proposed a more comprehensive criterion for determining MDSCs (Bronte et al., 2016). This criterion includes not only phenotypic properties but also functional and molecular characteristics. By taking into account various features of MDSCs, this proposal provides a standard for the definition and classification of MDSCs, which can help to reduce confusion in the characterization of MDSCs.

In glioma, MDSCs are expanded and recruited by a variety of inflammatory cytokines and chemokines secreted by glioma cells and other immune cells, such as GM-CSF (Horikawa et al., 2020), S100A8/9 (Gabrilovich and Nagaraj, 2009; Kwak et al., 2020), prostaglandin-E2 (PGE2) (Mao et al., 2014), CCL2 (Chang et al., 2016) and IL-8 (Alfaro et al., 2016). MDSCs are known to possess potent immunosuppressive capacity and promote the progression of glioma *via* multiple mechanisms. They can inhibit the activation and function of cytotoxic T cells through the production of various soluble factors such as ROS, NO, PGE2, IDO, IL-10, and S100A9 (Groth et al., 2019; Mi et al., 2020). Additionally, they can deplete metabolic substrates such as L-arginine to further suppress T cell function (Groth et al., 2019; Mi et al., 2020). MDSCs employ direct cell-to-cell contact as another mechanism to suppress the functions of effector T cells. The primary mode of MDSC-mediated inhibition towards T cells involves direct cell-to-cell contact through interactions such as PDL1/PD1, with soluble factors playing a secondary role (Bian et al., 2018; Groth et al., 2019). MDSCs also exhibit their suppressive potential indirectly by various ways such as recruiting regulatory T cells (Schlecker et al., 2012; Park et al., 2018), suppressing the activity of NK cells (Fortin et al., 2012) and interfering with antigen presentation function of dendritic cells (DCs) (Hu et al., 2011), and polarizing macrophages toward an anti-inflammatory M2 phenotype (Ostrand-Rosenberg et al., 2012). Therefore, MDSCs are an essential component of the immunosuppressive regulatory network in the glioma microenvironment, as they interact with multiple immune cells.

### 3.2 The interactions between OVs and MDSCs

While the immunosuppressive function of immature myeloid cells was acknowledged early on, the term “myeloid-derived suppressor cells (MDSCs)” was only introduced in a cancer context 15 years ago (Gabrilovich et al., 2007; Bronte et al., 2016). Consequently, there are limited studies on the interaction between OVs and MDSCs in glioma, and thus we broadened the scope of the review literature across any types of solid tumors beyond gliomas. **Table 2** shows that the majority of studies (18 out of 30) have reported an increase in MDSC infiltration in tumors following OV treatment, while a smaller number of studies (10 out of 30) have reported the opposite effect. Additionally, two studies have found that OV therapy has no significant impact on the proportion of MDSCs in local tumor regions. Notably, only three of these studies are specific to glioma (Otani et al., 2022). A recent study (Nguyen et al., 2022) showed that a third generation of adenovirus, Delta-24-RGDOX, elicits the resurface of an immunosuppressive tumor microenvironment that counteracts the tumor-killing effects of the virus. The activation of IDO, a critical immunosuppressive factor in OV infection, has been demonstrated to play a central role in promoting the frequencies of MDSCs and Tregs in the tumor microenvironment following OV treatment (Nguyen et al., 2022). Another study demonstrated that oncolytic HSV-1 (oHSV) not only recruits M2-macrophages but also MDSCs into the glioma microenvironment (Otani et al., 2022). Mechanistically, when glioma cells are infected with HSV-1, they activate Notch signaling in nearby uninfected glioma cells, as shown in a study by Otani et al. (Otani et al., 2022). Additionally, macrophages upregulate the expression of the Notch ligand, Jag-1, upon oHSV treatment, leading to CCL2 secretion and subsequent recruitment of MDSCs and M2 macrophages to the tumor site (Otani et al., 2022). In contrast, another study found that Newcastle disease virus (NDV) stimulates ‘immunogenic cell death’ (ICD) and ‘necroptosis’ in the GL261 glioma model, thereby increasing the infiltration of IFN-γ expressing CD4^+^ and CD8^+^ T cells while reducing the percentages of both PMN-MDSCs and M-MDSCs in the tumor microenvironment (Koks et al., 2015).

**Table 2:** The studies on the interactions between MDSCs and oncolytic viruses in various solid tumors.

Tumors	Oncolytic Viruses	Proportional change upon OV		Mechanisms involved in changes	Combination Treatment	Ref.
		MDSC	Other			
Colorectal tumor	HSV (HF10)	↑				(Esaki et al., 2013)
Peritoneal carcinomatosis	Reovirus	↑ (M)				(Clements et al., 2015)
Mesothelioma	MVTT	↑ (PMN)				(Tan et al., 2019)
Hepatocellular carcinoma	Newcastle Disease Virus (NDV)	↑	NK cells↑	STAT1, STAT3 activation	Fludarabine	(Meng et al., 2019)
Colon carcinoma	Sindbis Virus	→				(Scherwitzl et al., 2018)
Glioma	HSV	↑		Macrophages Jag-1↑→Notch↑→CCL2↑→ MDSCs recruitment	γ-secretase inhibitor (GSI)	(Otani et al., 2022)
Angiosarcoma	Sendai virus	↑	NK cells↑ Tregs↓ CD8+ T cells↑		IL-2	(Takehara et al., 2013)
Peritoneal carcinomatosis	Reovirus	↑			Gemcitabine	(Gujar et al., 2014)
Glioblastoma (GL261)	Newcastle disease virus (NDV)	↓	CD4+ T cells↑ CD8+ T cells↑			(Koks et al., 2015)
Lymphoma and melanoma	Reovirus	→		TLR3-dependent		(Katayama et al., 2018)
Colon cancer (MC-38)	Vaccinia viruses (IL-36γ-OVs)	vvTK: → vvTK-IL-36γ: ↓(G), →(M)	vvTK: TAMs→ Treg→ DC→ T cells→ vvTK-IL-36γ: TAMs↓ DCs↑ Tregs↑ NK cells↑ CD8+ T cells↑		IL-36γ armed OV	(Yang et al., 2021)
Colorectal cancer liver metastasis (CT-26)	HSV2	↓	Neutrophils↑, NK cells↑, T cells↑, B cells↑			(Zhang et al., 2021)
HPV-associated tumor (TC-1)	Newcastle disease virus (NDV)	↑	CD11b+ cells ↑			(Keshavarz et al., 2020)
Lymphoma	Vaccinia virus (OVV)	↑	CD8+ T cells↑ NK cells↑		Embelin	(Wang et al., 2020)
A mammary tumor (NBT1)	Vaccinia virus (VV-GMCSF)	↑			VV-neu (Recombinant Vaccinia HER2/neu)	(de Vries et al., 2015)
Liver cancer (Hepa1-6)	OVH-aMPD-1	OVH-aMPD-1:↑(pan), ↑(G), ↑(M) OVH: ↑(pan), →(G), →(M)			TIGIT antibody	(Lin et al., 2020)
Ewing sarcoma (A673)	HSV1 (rRp450)	↑	CD11b+ cells↑ TANs↑		Trabectedin	(Denton et al., 2018)
Colon cancer (MC38)	Vaccinia virus (vvDD-CXCL11)	↑(PMN) →(M)			α-PD-L1	(Liu et al., 2017)
Hepatocellular carcinoma	Newcastle disease virus (NDV)	↑		NDV-induced STAT3 activation, IDO1 upregulation, and MDSC infiltration	Dichloroacetate (DCA, a pyruvate dehydrogenase kinase (PDK) inhibitor)	(Meng et al., 2020)
Renal cancer (RENCA), mammary cancer (4T1), colon cancer (MC38)	Vaccinia virus (WR.TK-.Luc+)	4T1: ↑(Day 7), MC38: ↑ (Day 3, 7, 14), RENCA: →		COX2-mediated production of the prostaglandin PGE2 as a key determinant of MDSC tumor-infiltration	WR.TK-HPGD+ COX2 inhibitor celecoxib	(Hou et al., 2016)
Lung cancer (A549) Melanoma (B16)	Adenovirus (Ad5D24-CpG, Ad5D24)	→ (Ad5D24 vs. Ctrl) ↓ (Ad5D24-CpG vs. Ctrl)		CpG oligonucleotide blocks immune suppression by MDSCs	Ad5D24-CpG	(Cerullo et al., 2012)
Malignant peripheral nerve sheath tumor (MPNST) and neuroblastoma	HSV-1 (HSV1716)	↑(M) ↑(G)			Alisertib (Aurora A kinase inhibitor)	(Currier et al., 2017)
Sarcoma	HSV-1 (M002: IL-12 expressing HSV-1)	↓	CD4+ T cells↑, CD8+ T cells ↑, Activated monocytes ↑, Tregs ↑			(Ring et al., 2017)
Melanoma (B16.OVA)	Adenovirus (TILT-123: Ad5/3-E2F-d24-hTNFa-IRES-hIL2)	↓	M2↓, M1→, DCs→, Tregs→			(Cervera-Carrascon et al., 2021)
Colon adenocarcinoma (HCT-116)	Vaccinia virus (VACV)	↑				(Kilinc et al., 2016)
Peritoneal surface dissemination from colon cancer (PSD from CRC)	Vesicular stomatitis virus	↓	CD4+ T cells↑			(Day et al., 2020)
Colon cancer	HSV2	↓	Tregs↓ NK cells↑ CD8+ T cells↑ DCs↑			(Zhang et al., 2020)
Ovarian peritoneal carcinomatosis	Reovirus	↓	Tregs↓			(Gujar et al., 2013)
Pancreatic cancer	Vesicular stomatitis virus expressing Smac (VSV-S)	↓	Neutrophils↑ TAMs↓			(Tang et al., 2022)
Glioblastoma (GL261 and 005)	Adenovirus (Delta-24-RGDOX)	↑	Tregs↑	OV elicit IDO expression and activation	IDO inhibitor	(Nguyen et al., 2022)

↑, increased.

↓, decreased.

→, unchanged.

There is still no agreement on how OVs affect the proportion of MDSCs, but studies suggesting that OVs increase MDSC infiltration are becoming more prevalent. It is believed that the recruitment of MDSCs by OVs may depend on the type of virus, tumor, and the time of detection. The frequency of MDSCs in the tumor microenvironment changes over time after OV treatment. Early after viral infection, Ly6C^hi^ cells are recruited, which later transition into pro-inflammatory macrophages during the infection’s progression (Clements et al., 2017). It should be noted that there is variability in the definitions of MDSCs and gating strategies used across the studies included in **Table 2**, which may have contributed to the inconsistent conclusions.

The majority of studies suggest that OV-induced MDSC infiltration in glioma has a negative impact on the tumor-lytic effects of OV therapy. However, some studies suggest that OV action can reprogram MDSCs from a pro-tumor to an anti-tumor phenotype, despite an increase in MDSCs following OV treatment (Kilinc et al., 2016; Katayama et al., 2018). This reprogramming is believed to occur through mechanisms that increase NO production in MDSCs and inhibit immunosuppressive functions in a TLR3-dependent manner (Katayama et al., 2018). High levels of MDSC infiltration have been linked to the resistance of tumors to OV-mediated anti-tumor immune effects (Hou et al., 2016). The infiltration of MDSCs, therefore, appears to be a key determinant of OV resistance, which may not be overcome by increasing OV-mediated immune activation.

### 3.3 MDSCs-targeted therapy in combination with OV therapy

To improve the anti-tumor effect of OV therapy by overcoming the negative regulatory role of MDSCs, several research groups have implemented MDSC-targeted treatments (Shi et al., 2021). These strategies targeting MDSCs have been explored, including direct elimination, recruitment blockade, differentiation induction, and inactivation (Mi et al., 2020). Several ongoing or completed clinical trials, such as NCT04226066, NCT03294486, NCT02705196 (Bazan-Peregrino et al., 2021) have implemented 5-fluorouracil (5-FU) and gemcitabine, two chemotherapeutic drugs that have been demonstrated to selectively kill MDSCs in the glioma microenvironment. Due to their MDSC-depleting effects, these drugs exert synergistic anti-tumor effects when combined with OV therapy (Eisenberg et al., 2005; Esaki et al., 2013; Gujar et al., 2014). In studies involving OV therapy, reducing the recruitment of MDSCs has been a commonly used approach, with compounds and cytokines such as γ-secretase inhibitor (GSI) (Otani et al., 2022), IL-2 (Takehara et al., 2013), IL-36 (Yang et al., 2021), embelin (Wang et al., 2020), trabectedin (Denton et al., 2018), dichloroacetate (Meng et al., 2020), celecoxib (Hou et al., 2016) and indoximod (Nguyen et al., 2022) inhibiting MDSC infiltration through various mechanisms in the tumor microenvironment, thereby boosting the anti-tumor effects when combined with OV therapy.

## 4 Other immunosuppressive cells

While microglia, macrophages and MDSCs are the predominant cell types in the immunosuppressive microenvironment of glioma, the remaining immunosuppressive cells such as regulatory T cells (Tregs) and tumor-associated neutrophils (TANs) constitute a small fraction (Gieryng et al., 2017; Salemizadeh Parizi et al., 2021).

Tregs, a subpopulation of T cells, play a critical role in suppressing adaptive immune response through various mechanisms, such as upregulating immunosuppressive molecules (e.g., Foxp3, CTLA4, CD25, PD-1, and IDO), inhibiting antigen-presentation, secretion of immunosuppressive cytokines and consumption of IL-2 (Togashi et al., 2019; Bauer et al., 2021). Tregs are indispensable components in the immunosuppressive microenvironment of glioma, but it is unclear if OV therapy can alter their immunosuppressive function. Hypoxia and highly expressed HIF-1α is one of the main characteristics of glioma, especially glioblastoma. Yousaf et al. demonstrated that oncolytic virus therapy can reduce HIF pathway activity (Yousaf et al., 2020). However, Miska et al. found that the deficiency of HIF-1α in Tregs can actually enhance their immunosuppressive function and impede the anti-tumor immunity of CD8+ T cells in glioblastoma (Miska et al., 2019). This suggests that in the hypoxic environment of glioma, oncolytic viruses might enhance the immunosuppressive functions of Tregs by eliminating HIF-1α, even though hypoxia could also promote the replication of the virus (Reinblatt et al., 2004; Shayan et al., 2022). Nonetheless, more research is needed to determine the impact of oncolytic virus therapy on Tregs in the context of the hypoxic glioma microenvironment.

Currently, the interaction between OV infection and Tregs infiltration remains unclear. Several OV-related studies suggest that OVs can decrease the proportion of Tregs in the glioma microenvironment (Qiao et al., 2015; Saha et al., 2017; Todo et al., 2022). However, some reports present an opposite view. For example, Liu et al. found that a natural oncolytic alphavirus, M1, increases Treg cells in the tumor microenvironment of prostate cancer and melanoma. Targeting Tregs with CTLA4 antibody reduced the ratio of Treg/Teff and further enhanced the anti-tumor effect of OVs (Liu et al., 2021). Similarly, another study demonstrated that an oncolytic adenovirus, Delta-24-RGDOX, increases the frequency of Tregs in the glioma microenvironment, but this effect can be reversed by administering the IDO inhibitor indoximod to reduce the immunosuppressive microenvironment and the infiltration of Tregs (Nguyen et al., 2022).

More research is needed to fully understand the role of TANs in glioma virotherapy. the studies involving TANs have encountered a bottleneck as there are no clear molecular markers that distinguish them from mature neutrophils. While TANs and TAMs can both be classified into pro-tumoral N2 type and anti-tumoral N1 type, N2 overlaps with PMN-MDSCs both functionally and phenotypically (Bronte et al., 2016). Although the proportion of Tregs and TANs in the glioma microenvironment is small, they still have a significant impact on the immunosuppressive network that modulates OV-induced anti-tumor immune responses. Further research is therefore necessary to investigate the interactions between OVs and Tregs or TANs.

## 5 Concluding remarks

The success of OV therapy for glioma depends on the delicate balance between OV-induced anti-tumor immune responses and the presence of immunosuppressive cells. The interaction between OVs and immunosuppressive cells is complex, as OVs can recruit and alter the functions of immunosuppressive cells, while these cells can also affect OV-mediated immune responses. To optimize OV therapy, it is necessary to fine-tune the immunosuppressive cells in response to different OV-induced host immune reactions, maximizing anti-tumor immune responses and minimizing anti-viral immune responses (**Figure 2**). Recent approval of an oncolytic HSV-1 for glioma treatment in Japan has shed light on the potential of OV therapy. Despite the concerns of tolerance and economic burden caused by repeated stereotactic OV injection, serial administration of OV significantly enhances T cell-mediated anti-tumor immune response. It is likely counteracting the effects of innate immunosuppressive cells (Christie and Chiocca, 2022; Todo et al., 2022). In summary, the presence of immunosuppressive cells in the glioma microenvironment presents a significant challenge to the effectiveness of OV therapy. Therefore, it is crucial to explore ways to fine-tune this cell population in future basic and clinical research on glioma virotherapy.

## Author contributions

TL, JL, FY, and KS conceived the article. JL reviewed the literature, and JL, RP, and HN wrote the draft and prepared the figures and tables. All authors contributed to this article and approved the submitted version.

## Glossary

**Table:**

TAM	tumor-associated macrophage and microglia
MDSC	myeloid-derived suppressor cell
OV	oncolytic virus
TIL	tumor-infiltrating lymphocyte
CNS	central nervous system
BBB	blood-brain barrier
ICD	immunogenic cell death
TAA	tumor-associated antigens
PAMP	pathogen-associated molecular pattern
DAMP	damage-associated molecular patterns
HSV	Herpes Simplex Virus
AdV	Adenovirus
NDV	Newcastle Disease Virus
Treg	regulatory T cell
TAN	tumor-associated neutrophil
GM-CSF	Granulocyte-macrophage colony-stimulating factor
MIC-1	macrophage-inhibitory cytokine 1
MCP-1	Monocyte chemoattractant protein-1
CCL2/5/20	chemokine (C-C-motif) ligand 2, 5, 20
PMN-MDSC	polymorphonuclear MDSC
IDH	Isocitrate dehydrogenase
TMEM119	Transmembrane Protein 119
P2RY12	Purinergic Receptor P2Y
CD	cluster of differentiation
IL	interleukin
ATAC	Assay for Transposase-Accessible Chromatin
TGF-β	transforming growth factor β
IFN-γ	interferon-γ
TNF-α	tumor necrosis factor α
CTLA-4	cytotoxic T-lymphocyte-associated protein 4
PD-1	programmed cell death protein 1
FAS	Fas cell surface death receptor
DR5	death receptor 5
IDO	indoleamine 2,3-dioxygenase
DC	dendritic cell
CCN1	Cellular Communication Network Factor 1
STAT1	Signal Transducer And Activator Of Transcription 1
oHSV	oncolytic Herpes Simplex Virus
VV	Vaccinia Virus
VSV	Vesicular Stomatitis Virus
NK cell	natural killer cell
ROS	reactive oxygen species
NO	nitric oxide
GBM	glioblastoma multiforme
CL	clodronate liposomes
CPA	cyclophosphamide
CSF1R	colony stimulating factor 1 receptor
ICB	immune checkpoint blockade
M-MDSC	monocytic MDSC
HLA	human leukocyte antigen
TLR3	Toll-like receptor 3
5FU	5-fluorouracil
GSI	γ-secretase inhibitor
HIF-1	hypoxia-inducible factor 1
i.t.	intratumoral injection
DIPG	Diffuse Intrinsic Pontine Gliomas
ESIA	endovascular super-selective intra-arterial
CED	convection-enhanced delivery
TMZ	temozolomide
RT	radiotherapy
Pem	Pembrolizumab
HGG	high-grade glioma
IRES	internal ribosome entry site
